# Induction of posterior vitreous detachment (PVD) by non-enzymatic reagents targeting vitreous collagen liquefaction as well as vitreoretinal adhesion

**DOI:** 10.1038/s41598-020-64931-3

**Published:** 2020-05-19

**Authors:** Mithun Santra, Maryada Sharma, Deeksha Katoch, Sahil Jain, Uma Nahar Saikia, Mangat R. Dogra, Manni Luthra-Guptasarma

**Affiliations:** 1 Department of Immunopathology, Postgraduate Institute of Medical Education and Research, Chandigarh, 160012 India; 2 Present Address: Department of Otolaryngology and Head & Neck surgery, Postgraduate Institute of Medical Education and Research, Chandigarh, 160012 India; 3 Department of Ophthalmology, Postgraduate Institute of Medical Education and Research, Chandigarh, 160012 India; 4 Department of Histopathology, Postgraduate Institute of Medical Education and Research, Chandigarh, 160012 India

**Keywords:** Drug development, Preclinical research, Integrins

## Abstract

Induction of posterior vitreous detachment (PVD) by pharmacologic vitreolysis has been largely attempted through the use of enzymatic reagents. Ocriplasmin has been the only FDA-approved clinical reagent so far. Several adverse effects of ocriplasmin have emerged, however, and the search for alternative PVD-inducing reagents continues. Since i) collagen forms an important structural component of the vitreous, and ii) strong vitreo-retinal adhesions exist between the cortical vitreous and the internal limiting membrane (ILM) of the retina, an effective PVD-inducing reagent would require both, vitreous liquefaction, and concurrent dehiscence of vitreoretinal adhesion, without being toxic to retinal cells. We designed a combination of two reagents to achieve these two objectives; a triple helix-destabilizing collagen binding domain (CBD), and a fusion of RGD (integrin-binding) tripeptide with CBD (RCBD) to facilitate separation of posterior cortical vitreous from retinal surface. Based on *in vitro*, *ex-vivo*, and *in vivo* experiments, we show that a combination of CBD and RCBD displays potential for safe pharmacologic vitreolysis. Our findings assume significance in light of the fact that synthetic RGD-containing peptides have already been used for inhibition of tumor cell invasion. Proteins such as variants of collagen binding domains could have extended therapeutic uses in the future.

## Introduction

The vitreous humor is a hydrogel present in the eye; an intact vitreous gel is important for ocular health. However, with aging, the gel undergoes a gradual, spontaneous process of liquefaction, and separates from the retina, resulting in posterior vitreous detachment or PVD^1–3^. Therefore, it is not surprising that there is a high incidence of vitreo-retinal disorders in later decades of life, with the peak of incidence of retinal conditions matching the peak age of incidence of posterior vitreous separation^4–6^. When the gel liquefaction exceeds the extent of vitreo-retinal dehiscence, anomalous PVD occurs, which is characterized by partial or incomplete detachment of vitreous from the retina. Anomalous PVD can involve exertion of excessive tractional forces acting upon the retina, which can lead to many ophthalmic complications such as hemorrhage, retinal tears, or detachment, macular hole formation, and vitreomacular traction syndrome^3,7,8^.

The mainstream treatment of incomplete PVD is surgical intervention or vitrectomy, to create a clean retinal surface devoid of any vitreous remnants and thus avoid further complications such as proliferative vitreoretinopathy (PVR). However, owing to its invasive nature and associated risks, surgical vitrectomy is not an ideal choice, except in cases complicated by retinal detachments. In paediatric patients, induction of posterior vitreous detachment (PVD) is an important and challenging step in the successful management of retinal detachment or traumatic macular hole, owing to the firm vitreoretinal adhesions. Pharmacological vitrectomy has been explored as a non-surgical, safer and cleaner alternative to surgical vitrectomy, or as an adjunct before or during vitrectomy to facilitate the induction of PVD. The pharmacological reagents thus far attempted for vitreolysis are enzymatic (such as bacterial collagenase, hyaluorinidase, dispase, plasmin and ocriplasmin) or non-enzymatic (such as vitreosolve and RGD based peptides)^9^. Of the several enzymatic reagents, the plasmin-derived ocriplasmin has been the only reagent approved by the FDA. However, there are safety concerns associated with its use, owing to its non-specificity and resultant side effects. Non-enzymatic reagents developed to achieve vitrectomy offer the added benefit of safety without any collateral damage to the retinal structures. In this direction, the RGD (Arg-Gly-Asp) containing peptides have been used to induce PVD^9,10^.

Previous attempts to use bacterial collagenase as a pharmacovitreolytic reagent did not meet with much success^11–15^. This is because the collagenase is highly active, leading to toxicity in the context of vitreolysis. Among the many collagenases of bacterial source, the *Vibrio mimicus* collagenase or VMC is the least explored. VMC has a very distinct collagen binding domain (CBD) containing two FAXWXXT motifs which are understood to be essential for its activity^16^.

**Figure 1:**
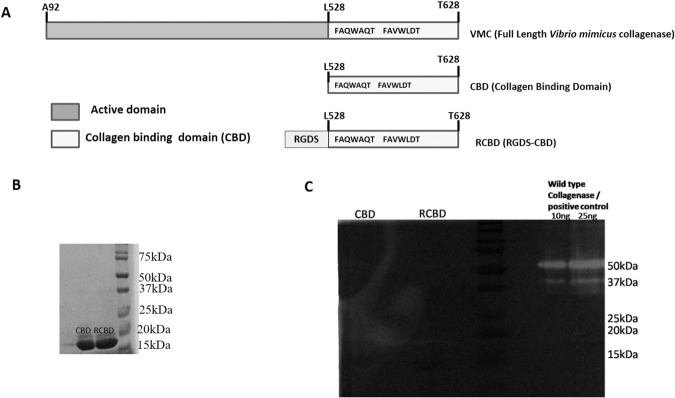
(Panel A) Schematic representation of vibrio mimicus collagenase (VMC)-derived constructs; (Panel B) SDS-PAGE profile of the proteins, CBD and RCBD; (Panel C) Zymography showing no gelatinolytic activity of CBD or RCBD. Full-length gel is presented in Supplementary Fig. S2.

Since collagen is the major contributor to ocular viscoelasticity and vitreoretinal adhesions, and interlinking collagen fibrils along with Hyaluronic Acid (HA) form the gel-like consistency of vitreous^3^, we chose to use the collagen-binding domain (CBD) motif derived from *Vibrio mimicus* collagenase (VMC) (Fig. 1A) for this purpose. Unlike collagenases of eukaryotic origin, the bacterial collagenases (such as *VMC*) have the catalytic activity domain and the collagen binding domains separated from each other. This makes it possible to clone and purify the CBD independent of the active domain. We hypothesized that instead of using the whole collagenase molecule, which can be toxic to the ocular tissues, we could use the CBD domain alone. The rationale of using CBD domain was that the CBD would bind to the collagen triple helix and cause its destabilization (without collagen degradation) potentially by loosening up the triple helical structure of collagen, as has been demonstrated in a non-ophthalmological context^17^. This is the first attempt at using CBD (rather than the whole collagenase molecule) in inducing PVD. The CBD was subsequently fused to an RGD (Arg-Gly-Asp) motif (hereafter referred to as RCBD) in an effort to facilitate PVD. The addition of the RGD motif to CBD was done to promote competition for binding sites on integrins (which normally bind to components of the ECM)^18^, and thereby disrupt integrin-ECM interactions, causing reduction in vitreoretinal interaction at the Inner Lining Membrane (ILM) of the retina. Our data shows that together, the combination of CBD and RCBD can cause PVD through structural perturbation of the collagen fibrils, as well as through decreased adhesion of collagen with the cells of the ILM.

## Results

### Cloning, expression and purification of CBD and RCBD

Figure 1A shows the diagrammatic representation of the full length *vibrio mimicus* collagenase (VMC); the collagen binding domain forms a part of the VMC. The design of the fusion of the tripeptide, RGD (Arg-Gly-Asp) with CBD (i.e., RCBD) is also shown. Following cloning of genes for CBD and RCBD (Supplementary Fig. S1), the proteins were expressed and purified (Fig. 1B; Supplementary Fig. S2); zymography revealed that CBD and RCBD have no gelatinolytic activity, unlike the collagenase, VMC, from which these are derived (Fig. 1C).

### Stability of CBD and RCBD

The stability of CBD and RCBD as well as combination of CBD and RCBD was tested over 48 hours of incubation in the presence of cadaver-derived vitreous; it was observed in each case, that more than 80% of the proteins remained intact and were not degraded over this time period (Supplementary Fig. S3A,B).

### Retinal cell viability upon exposure to CBD/RCBD

In order to determine the dose of CBD and RCBD to be used in vitreolysis experiments, MTT assay was carried out. Both, CBD as well as RCBD did not show any toxicity on ARPE-19 cells, even up to a concentration of 30 µM (Supplementary Fig. S3C).

### Structural analysis of VMC variants with circular dichroism spectroscopy

**Figure 2:**
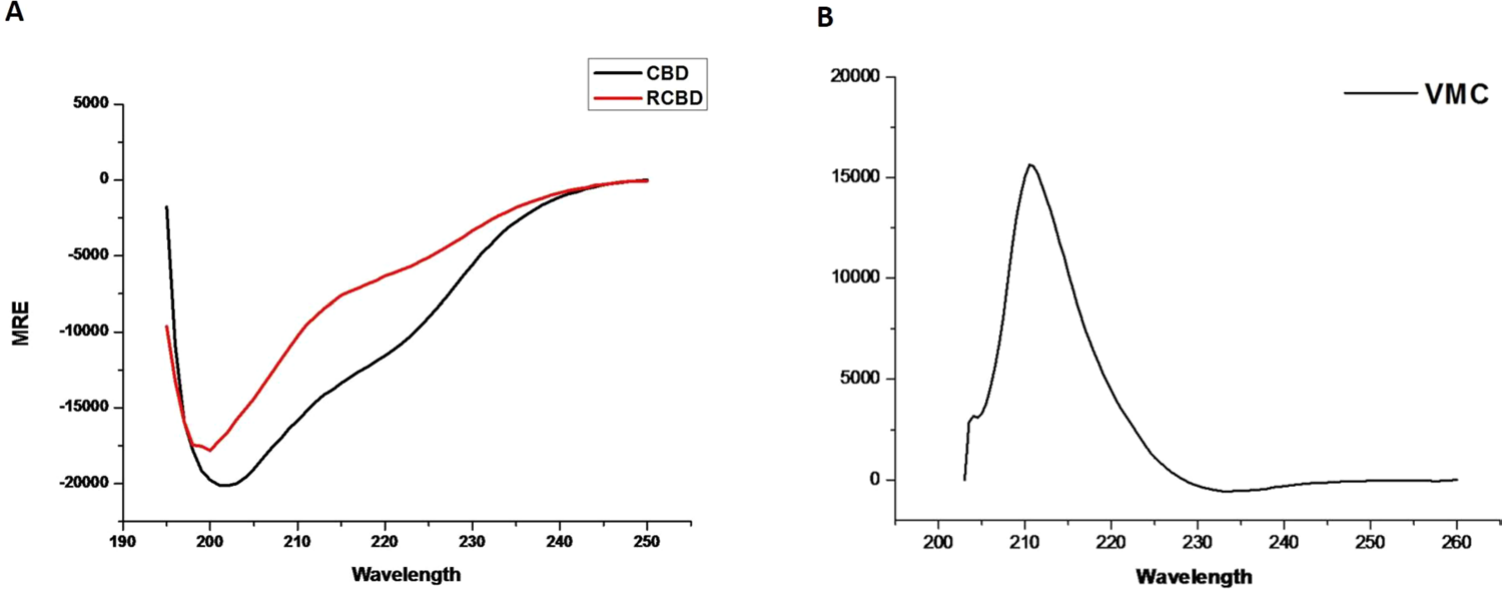
Secondary structure determination by circular dichroism (CD). CD spectra of CBD and RCBD (Panel A) and VMC (Panel B).

Circular dichroism (CD) was carried out to assess the structural signatures of CBD and RCBD in comparison with VMC (which carries the active domain of the enzyme in addition to the collagen-binding domain). CD spectra of all three proteins (CBD, RCBD, and VMC) were recorded; as shown in Fig. 2, both, CBD as well as RCBD show the characteristic α-helical structural content indicated by bands at 222 and 208 nm, whereas, VMC shows the characteristic positive ellipticity at 210 nm^19^.

### Circular dichroism (CD) spectroscopy shows alteration of collagen triple helix structure upon CBD and RCBD treatment

Studies suggest that mammalian as well as bacterial collagen-binding domains may have some helicase or collagen unwinding activity^17,20^; various collagenases unwind the collagen triple helix through the binding of CBD to the collagen triple helix, prior to hydrolysis^17^. The collagen helix unwinding effect of the CBD of *Vibrio mimicus* collagenase (VMC) has not been explored yet. In order to investigate whether the CBD of VMC may also have similar collagen helix unwinding activity, CD spectroscopy was performed.

Collagen triple helix has a distinct sinusoidal spectrum with a positive peak at 221 nm and negative peak at 198 nm^21,22^. The intensity of the positive peak at 221 nm (representing the collagen triple helix region) decreases upon denaturation^19,23^. It is to be expected that if binding of CBD to the collagen helix results in loosening/unwinding of the helix, it will lead to changes in CD spectra as compared to the spectra of collagen alone. Such changes in CD spectra have also been reported earlier in the case of binding of the collagen-binding domain of mammalian collagenase to collagens^24^.

**Figure 3:**
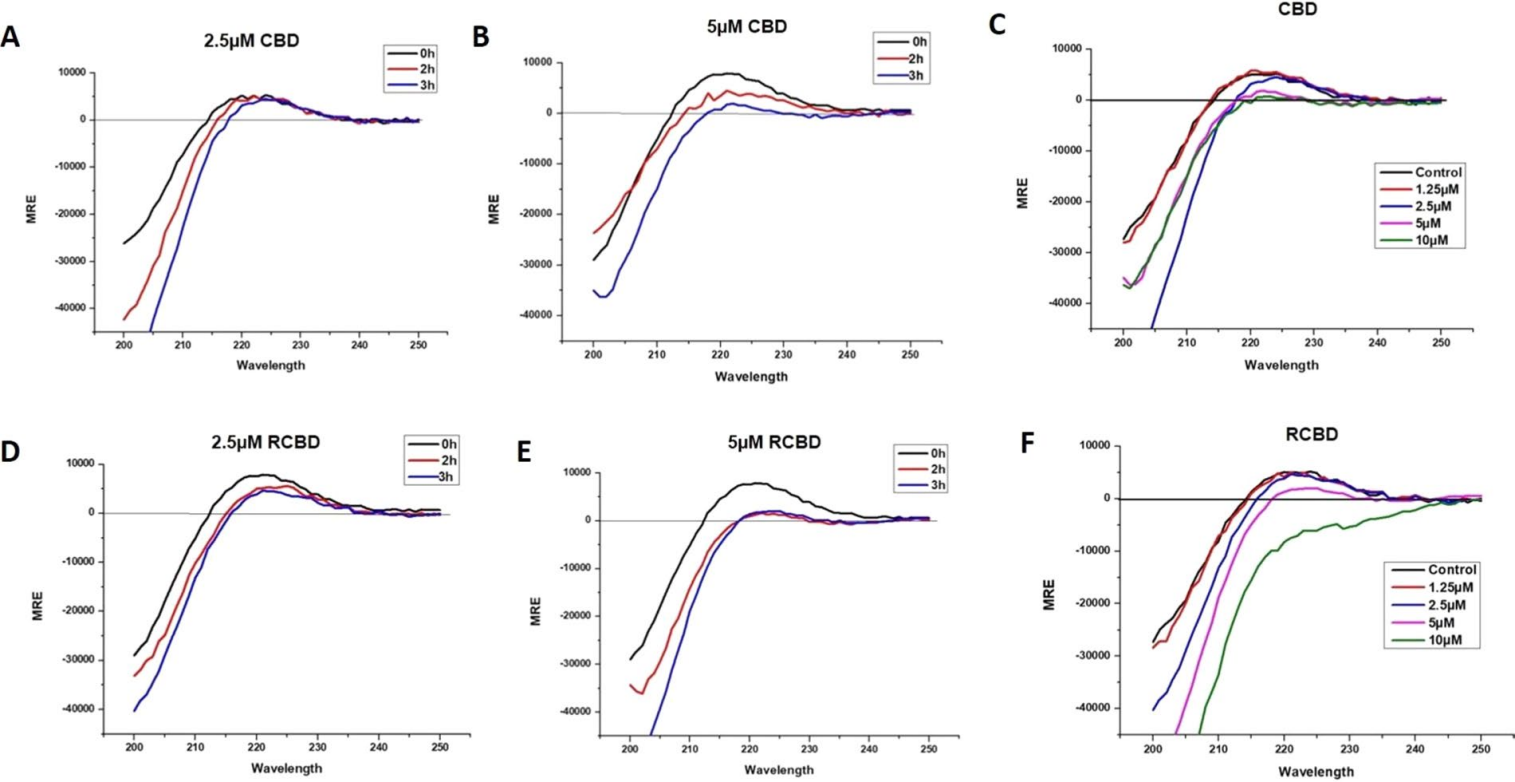
Structural perturbation of collagen upon binding of CBD and RCBD. Mean residue ellipticity (MRE) vs wavelength plots for collagen type I incubated with CBD (Panels A-B) and RCBD (Panels D-E) at two different concentrations (2.5 µM and 5 uM) at 30 °C, for 0 h, 1 h and 3 h; similar plots for col I incubated with varying concentrations of CBD (Panel C) and RCBD (Panel F) at 30 °C after 3 h of incubation with the respective reagents.

As shown in Fig. 3, CD spectra showed a decrease in signal of positive peak of collagen (221 nm) upon treatment of collagen with CBD as well as RCBD, in a time-dependent as well as dose-dependent manner. A concentration of 2.5 µM of CBD was ineffective in causing structural changes in collagen triple helix, unlike RCBD (at the same concentration) which was effective after 2 h of incubation. CBD was effective at concentration above 5 µM. These results suggest that both of these reagents may unwind the collagen triple helix, resulting in the decrease of positive peak at 221 nm. Therefore, in the context of vitreolysis, CBD or RCBD may destabilize the collagen fibrils network and loosen up (liquefy) the vitreous gel.

### CBD and RCBD can solubilize polymerized collagen

**Figure 4:**
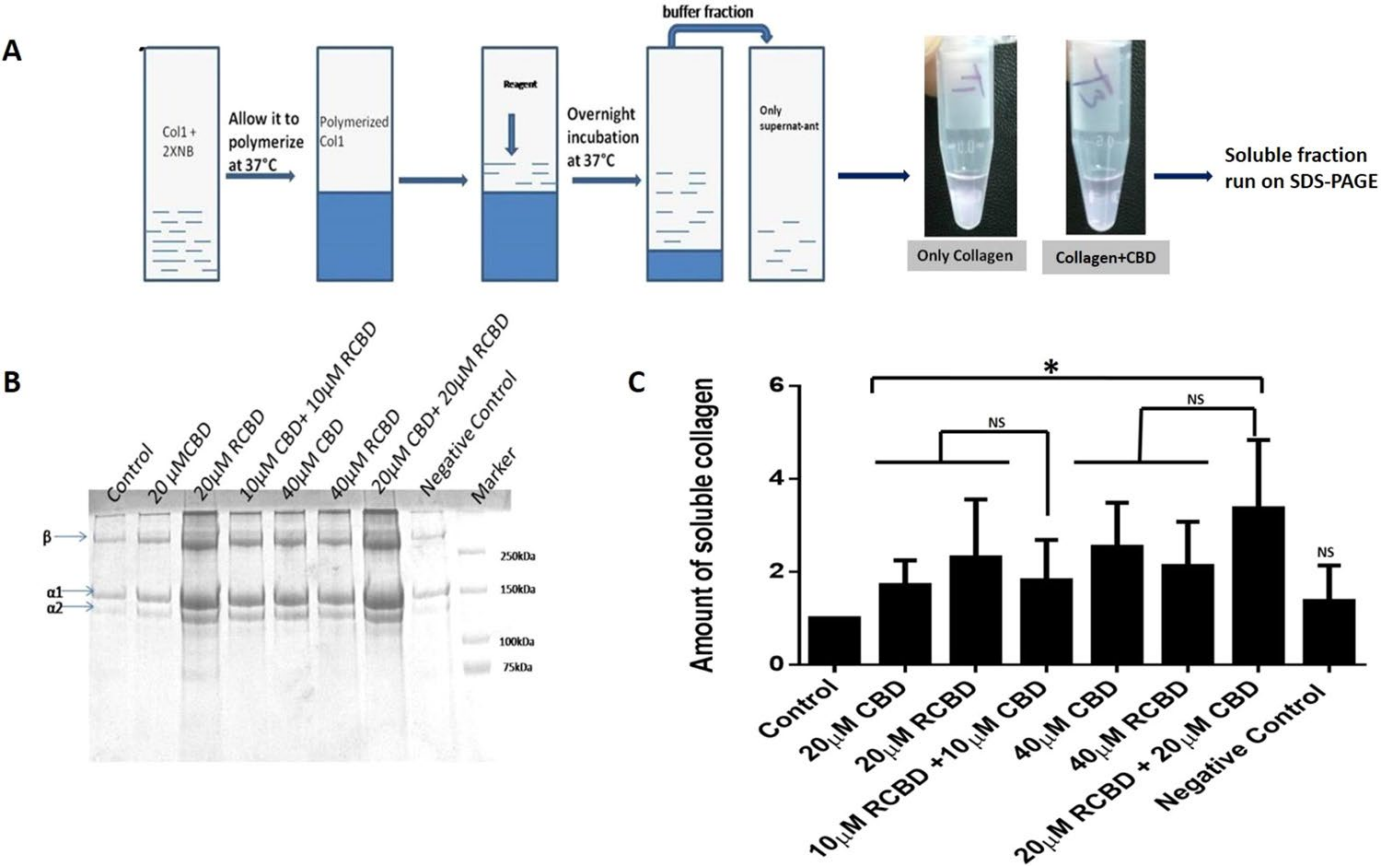
Collagen solubilization assay. **(**Panel A) Experimental outline of the protocol; collagen type 1 was polymerized in the presence of neutralizing buffer, followed by treatment with the test reagents, and incubated overnight at room temperature; the supernatant from each tube was run on SDS-PAGE to assess the released monomeric soluble collagen. A representative image showing decreased opacity of the polymerized collagen in the presence of CBD as compared to the control tube (minus CBD); (Panel B) Representative SDS-PAGE gel of the assay; (Panel C) Upon treatment with CBD/RCBD, polymerized collagen releases the monomeric form of (soluble) collagen, seen to be increased in samples treated with reagents (indicated by increased band intensity of α1 and α2 chains of collagen), as compared to untreated sample of collagen (control). Negative control: 20 μM CK (cysteine knot domain in the C-terminal region of CTGF); Data represents the mean (± standard deviation, SD) of five independent experiments (*p < 0.05, significant; NS, Not Significant).

In order to further confirm the ability of CBD and RCBD to unwind the collagen triple helix, commercially sourced collagen type 1 was polymerized in the presence of neutralizing buffer. Although collagen type II is the primary collagen present in the vitreous, it is known that collagen types I, II and III have similar structure^25^. Collagen solution was treated with two different concentrations (20 µM and 40 µM) of CBD and RCBD individually, and a combination of both proteins, CBD and RCBD (10 µM CBD and 10 µM RCBD; 20 µM CBD and 20 µM RCBD) for a period of 12 h. The supernatant from each tube was assessed on SDS-PAGE to examine the released monomeric soluble collagen (signifying liquefaction of collagen), in terms of increased intensity of collagen bands (α1, α2, β) observed in presence of reagents in comparison with collagen in the absence of any added reagent (control) (Fig. 4A). For quantitative analysis, the α1 bands corresponding to untreated and treated collagen samples were measured densitometrically using ImageJ software (https://imagej.nih.gov/ij/; NIH, USA), and the amount of soluble collagen was plotted as the ratio of intensity of α1 band in treated vs control samples.

Polymerized collagen partially mimics the gel-like vitreous. The amount of soluble collagen released from triple helix by CBDs is expected to be directly proportional to the triple helix unwinding ability of CBD. Our data suggests that upon treatment with CBD and RCBD, the polymerized collagen loosens up and is released in the soluble phase, which is detected in SDS-PAGE in the form of increased band intensity of α1 and α2 chains of collagen. Collagen band (α1, α2, β) intensity was observed to increase in a dose-dependent manner (Fig. 4B). The amount of monomeric collagen released in the presence of CBD and/or RCBD was significantly higher as compared to that seen with untreated collagen gel (Fig. 4C). It may be noted that the combination of CBD and RCBD did not cause any significant increase in collagen solubilization when compared with the solubilization resulting from presence of either reagent used independently (Fig. 4C).

### RCBD binds to cell surface integrins

**Figure 5:**
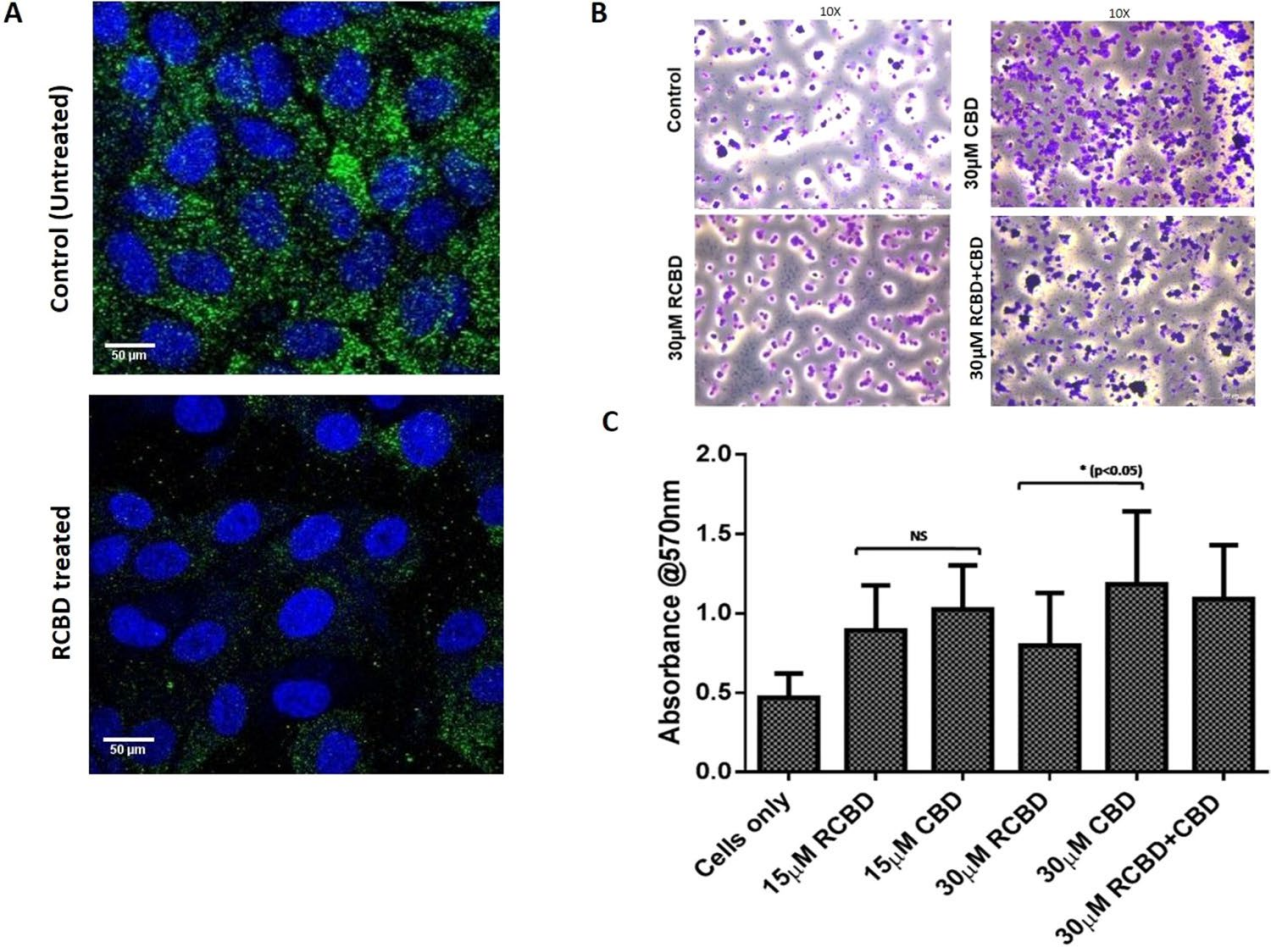
RCBD binds to cell surface integrins and causes decreased adherence of ARPE-19 cells to collagen as compared to CBD: (Panel A) Immunofluorescence staining of β1 integrin in ARPE-19 cells. Cells were seeded on coverslips in presence of vitreous derived from patients undergoing surgery for retinal detachment, in the absence and presence of RCBD; immunofluorescence staining was done using mouse anti-β1 integrin antibody and goat anti-mouse-FITC secondary antibody. β1 integrin-associated fluorescence is shown in green and nuclei (DAPI) in blue; scale bar: 50 µm (Panel B) Adherence of ARPE-19 cells (untreated or pre-treated with CBD and/or RCBD) on collagen coated surface; adhered cells were imaged; scale bar: 400 µm, 10X magnification; and (Panel C) Bar diagrams represent the corresponding quantitative data.

In order to test whether the RGD motif present in RCBD has the ability to bind to cell surface integrin receptors, we cultured ARPE-19 cells in the presence of pathologic vitreous, in the absence and presence of RCBD protein, followed by staining of cells with anti-integrin antibody. As shown in Fig. 5, the ARPE-19 cells express β1 integrin receptor when cultured in the presence of vitreous; when cells were cultured in the presence of RCBD, there was decreased binding of the antibody to the integrin receptors, suggesting that the RGD motif (in RCBD) is available to bind to the integrins, causing inhibition of binding of the anti-integrin antibody.

### RCBD inhibits attachment of ARPE-19 cells to collagen

RGD peptide is a well-known inhibitor of integrin-mediated interaction of cells with the extracellular matrix (ECM). To evaluate the efficacy of RCBD (RGD-CBD)-mediated inhibition of attachment of cells with collagen-coated surface, adhesion of ARPE-19 cells was assessed on collagen type 1 coated surface, in the presence of CBD as well as RCBD.

Data from the adhesion experiment showed that 30 µM RCBD was effective in significant reduction of adhesion of ARPE-19 cells to collagen-coated surface as compared to cells treated with 30 µM CBD (Fig. 5B,C). It may be noted that as expected, cells treated with CBD alone showed increased binding to collagen as compared to untreated cells, due to its ability to bind to collagen, causing unwinding of the helical structure, and consequent exposure of integrin-binding motifs that can bind to cells. A detailed analysis of these observations is discussed later (under “Discussion”).

### *Ex-vivo* experiments for assessment of vitreolysis: Rheology, OCT and SEM

#### Rheological studies

**Figure 6:**
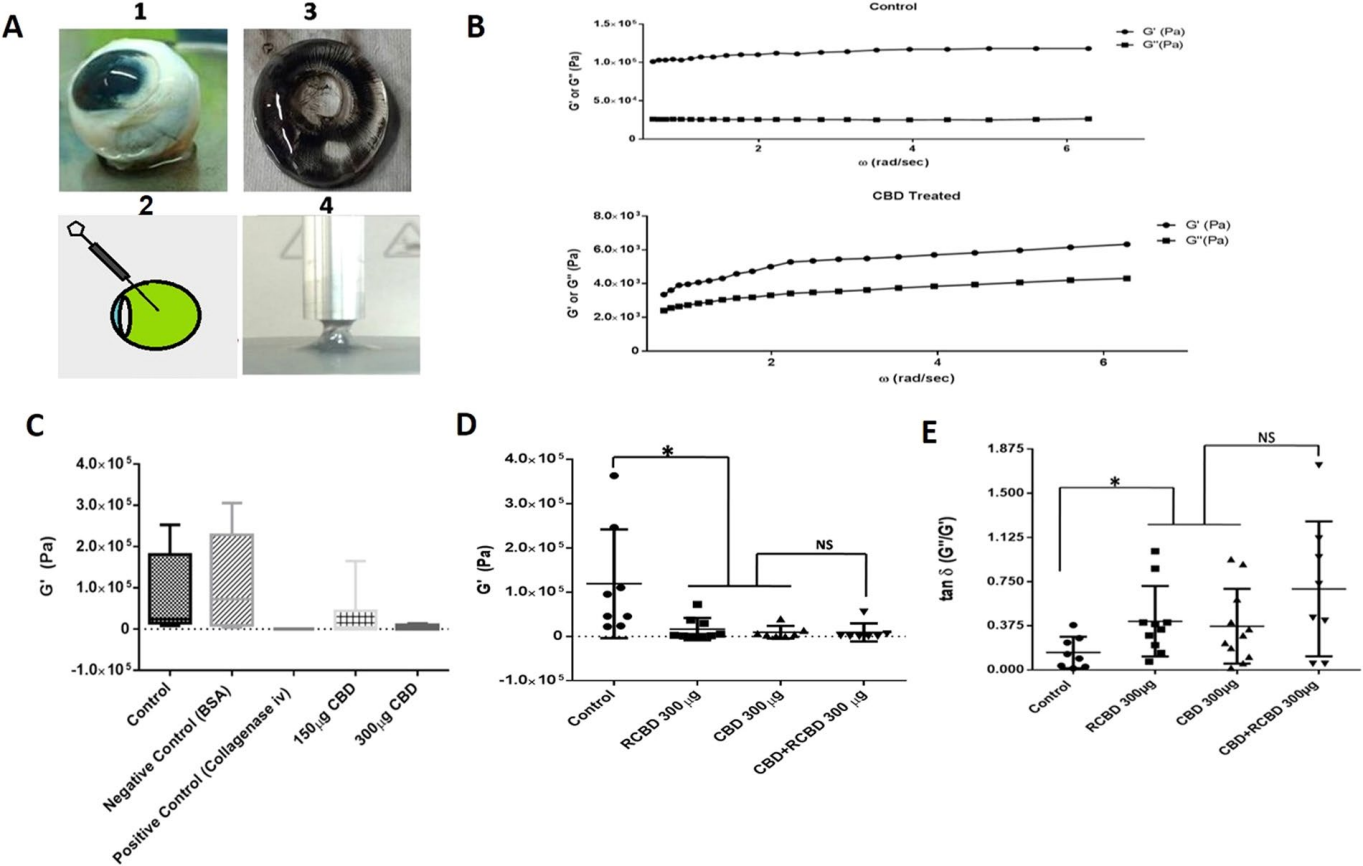
Rheological studies of goat eye vitreous treated with CBD/RCBD vitreous: (Panel A) Experimental set-up; (Panel B) Measurements of storage modulus (G’) and loss modulus (G”) were done for the control vitreous sample (treated with PBS) and vitreous treated with reagents. Frequency sweeps were recorded at fixed shear strain amplitude (γ○ =3%); linear viscoelastic region (i.e., modulus independent of frequency) is taken within the range of π/5 to 2π rad/s of oscillatory frequency sweep. Error bars indicate standard deviation; (Panel C) Upon treatment of vitreous with CBD, G’ decreased in a dose-dependent manner whereas injection with PBS (control) or BSA (negative control) did not show any change in G’; (**D**) Both, CBD as well as RCBD-treated vitreous resulted in a significant decrease in storage modulus (P < 0.05). The combination of CBD and RCBD showed a similar effect as seen with individual reagents; (**E**) The loss tangent (G”/G’) increased significantly in the presence of either reagent used individually, as well as in combination, as compared to control (p < 0.05). (**No of goat eyeballs per treatment group, n = 3).

In order to test the liquefaction of vitreous in goat eye balls, reagents were injected into the vitreous cavity (Fig. 6A); control eyes received an intravitreal injection of PBS alone (control). Following intravitreal injection, eyeballs were incubated for 5 h, followed by rheological studies.

The shear strain amplitude (γ_0_) was set at 3% with an oscillatory frequency sweep, ω = π/5 to 2π rad/sec, to obtain a linear viscoelastic behaviour^26^. The linear viscoelastic region was determined by measuring storage modulus (G’), loss modulus (G”) and dumping factor or loss factor (tan δ = G”/G’) (Fig. 6B).

Upon addition of CBD or RCBD to goat vitreous, the storage modulus or G’ was observed to have decreased, when compared with the control (vitreous injected with PBS alone); as expected, the negative control (BSA-injected) behaved similar to that of control vitreous, and the value of G’ decreased with increase in concentration of CBD (Fig. 6B,C). The combination of CBD and RCBD (150 µg CBD + 150 µg RCBD) had a similar impact on G’ as seen when CBD or RCBD were used independently (300 µg CBD or 300 µg RCBD) (Fig. 6D). The ratio between the loss and storage modulus in a viscoelastic material (defined as tan δ) is a measure of alteration in macromolecular structure; treatment with CBD, RCBD or a combination of CBD and RCBD, resulted in a significant increase in tan δ in the presence of each of these reagents, as compared to the control (Fig. 6E).

#### Evaluation by OCT and SEM

**Figure 7:**
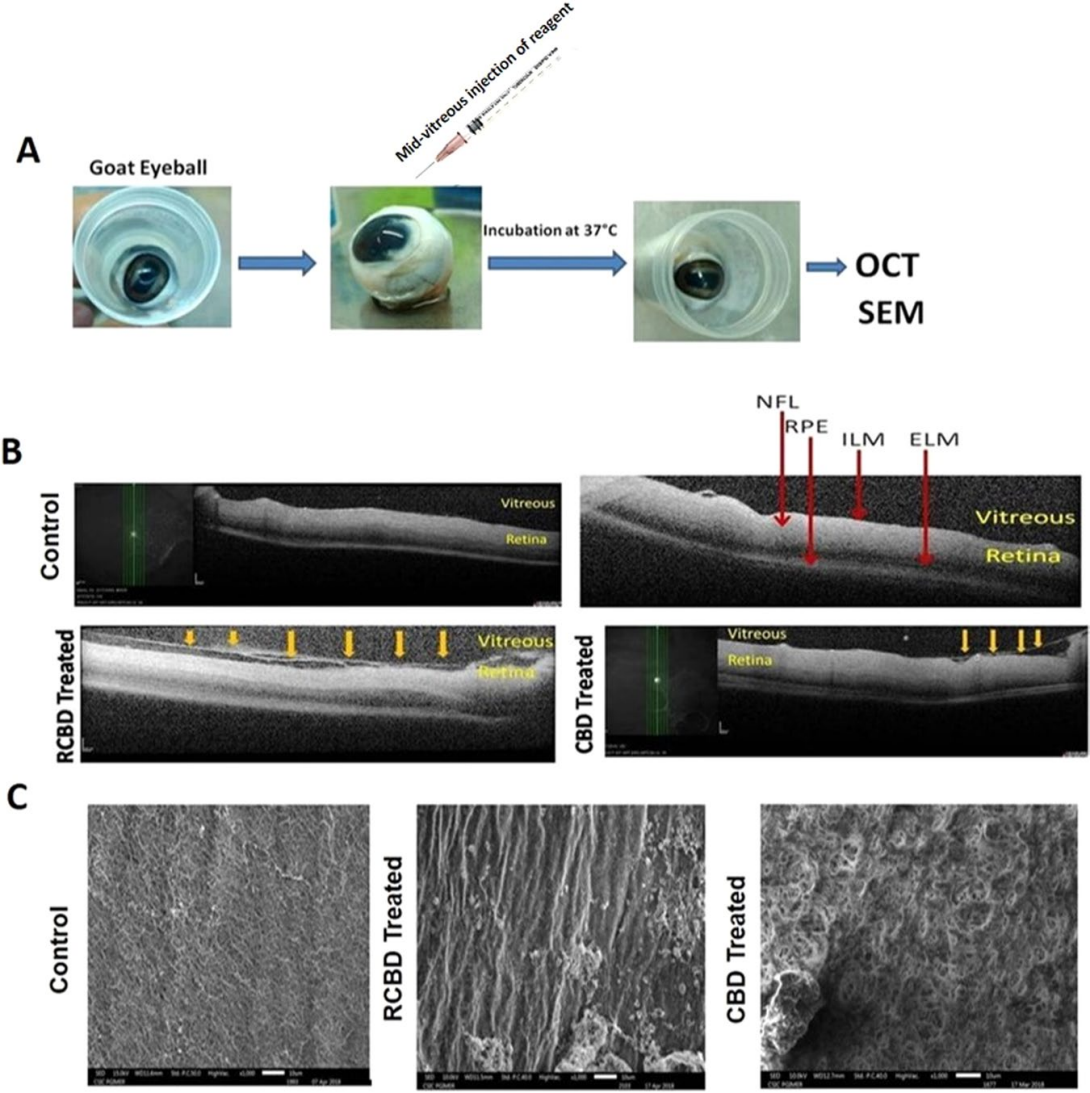
*Ex-vivo* OCT and SEM studies in goat eyeballs: (Panel A) Schematic representation of the experimental outline; (Panel B) Representative OCT images showing detachment of posterior hyaloid membrane of vitreous from the ILM of the retina upon treatment with 250 µg CBD or 350 µg RCBD (shown by arrows); no posterior vitreous detachment (PVD) was seen in the corresponding contralateral eyes; (Panel C) SEM micrographs showing dense vitreous fibrils on the retinal surface of control eye, which were cleared/disorganized upon treatment with reagents (**scale bar: 10 µm); (ELM: External Limiting Membrane, ILM: Inner Lining Membrane, NFL: Nerve Fiber Layer, RPE: Retinal Pigment Epithelial cells).

Following intravitreal injection of CBD and/or RCBD in goat eye balls, the occurrence of PVD was examined by OCT and SEM (Fig. 7A).

OCT examination showed that upon treatment with CBD and RCBD, there was a separation of the posterior cortical layer of vitreous from the ILM of the retina (highlighted by arrows in Fig. 7B), whereas control eyes showed no such detachment.

Scanning electron microscopy (SEM) showed dense vitreal collagen fibrils on the inner surface of retina or ILM, with intact vitreoretinal adhesion on the retinal surface in the control eye, in the absence of any reagents; treatment with CBD and RCBD caused a disruption of the molecular structure of vitreal collagen (Fig. 7C). The effective dose of CBD and RCBD required to effect PVD was 75 µg per ml and 60 µg per ml of vitreous respectively.

### Validation of posterior vitreous detachment by combination of CBD and RCBD in rabbits

This part of the study was conducted with 10 New Zealand rabbits, which received a combination dose of CBD & RCBD (150 µg each) in the left eye, while the contralateral eye (right eye) was injected with PBS, and referred to as control eye. A brief experimental outline is given in Supplementary Fig. S5.

#### Clinical evaluation (Indirect ophthalmoscope and B-ultrasonography)

Indirect ophthalmoscopic evaluation did not find any abnormalities in either of the two eyes of all animals before intravitreal injection. All eyes had a clear media without any visible membrane in the vitreous prior to injection.

Media clarity was graded as 1 when optic disc and retinal/choroidal vessels were clearly seen upto the periphery, grade 2 when the optic disc and second order vessels could be seen, grade 3 when the optic disc and major vessels around the disc only could be seen and grade 4 when the optic disc was not visible.

**Figure 8:**
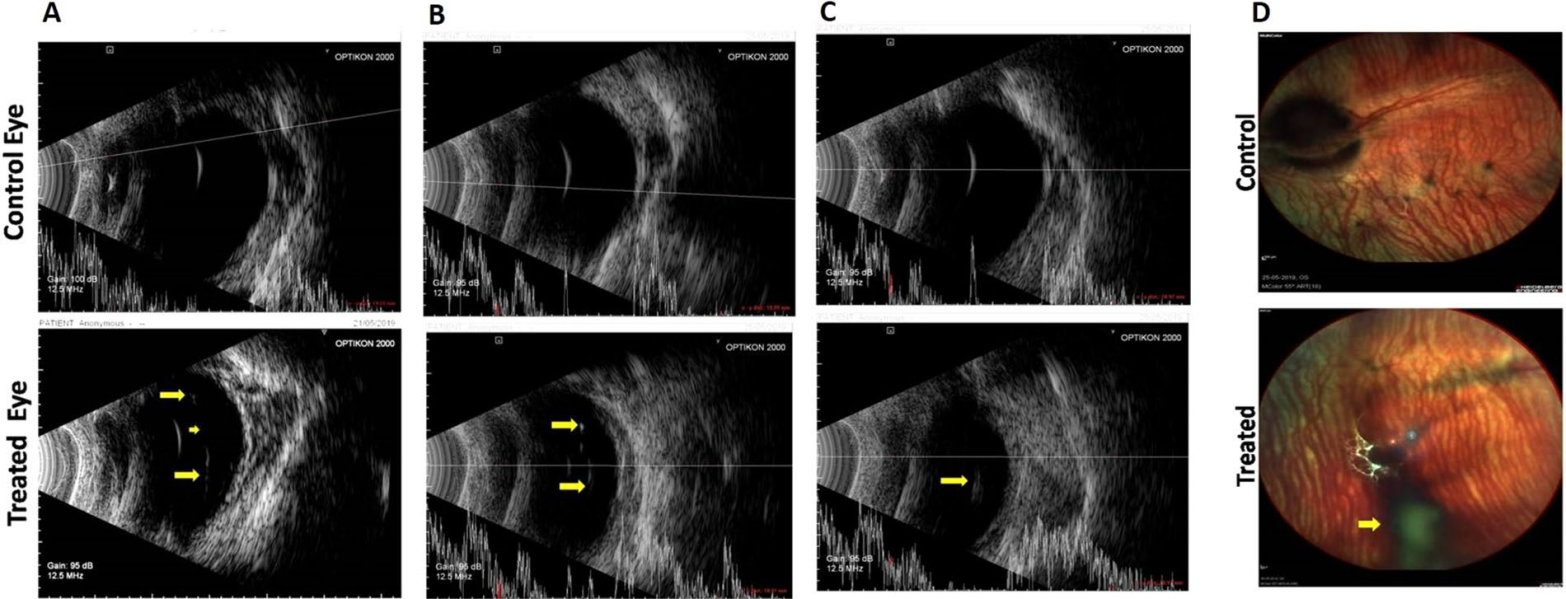
B-scan ultrasonography in three representative eyes following treatment with RCBD + CBD: Representative images of eyes treated with CBD and RCBD showing complete PVD (Panels A and B, marked by arrows), and partial PVD (Panel C), along with the corresponding contralateral eyes (controls). (Panel D) Fundus of representative rabbit eye with complete PVD, following the intravitreal injection of combination of CBD and RCBD; the posterior hyaloid membrane appears as a shadow in the fundus image which reflects PVD (marked with arrow).

Post injection, after 24 h, vitreous was still clear in most of the treated eyes, except rabbits 1 and 2 which showed a mild vitreous haze; however, in these eyes too, the vitreous cleared up by day 8. There were no unwanted abnormalities like vitreous or retinal haemorrhage, retinal edema, retinal detachment, or any effect on the lens in any of the control or treated animals (Supplementary Table 1). It was found that in only one animal (rabbit number 3), vitreous had media grade 2 on day 4 and remained like this till day 8. The presence of fibrin in the anterior chamber in some animals resolved with time (i.e., by day 8). Treatment with the reagents led to a floating posterior hyaloid membrane as seen by indirect ophthalmoscope in eyes with complete PVD, whereas, in control eyes, there was no such detachment. B-scan ultrasonography demonstrated a complete (Fig. 8A,B) or partial (Fig. 8C) detachment of the posterior hyaloid upon intravitreal injection of reagents, whereas in control eyes (PBS), there was no evidence of vitreous detachment. On 8th day, multicolor fundus pictures revealed no signs of retinal toxicity, vitreous opacity, retinal detachment, or haemorrhage, and in eyes with complete PVD, the posterior hyaloid membrane formed a shadow due to PVD, seen in the image as a dark spot (Fig. 8D).

#### Scanning electron microscopy

**Figure 9:**
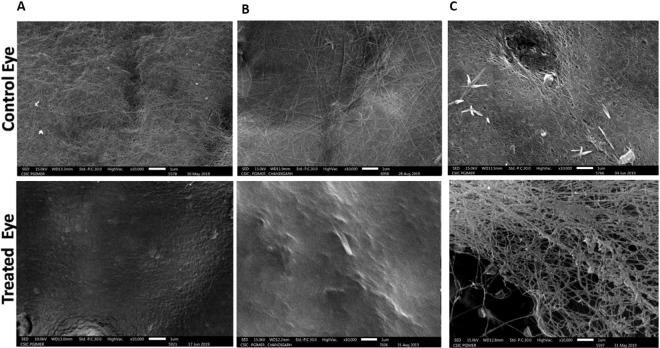
Scanning electron photomicrographs of the inner retinal surface of 3 representative animals treated with CBD and RCBD. In test eyes with complete PVD (Panels A and B), the retinal surface was devoid of any collagen fibers; in eyes with partial PVD (Panel C), there was less vitreous cortex left on the retinal surface, compared to the corresponding control (contralateral) eyes (PBS) with no PVD, where dense vitreous cortical fibers were visible (** Scale Bar 1 µm or 10000X magnification).

Scanning electron microscopy showed dense vitreal collagen fibrils on the inner surface of retina or ILM, with intact vitreoretinal adhesion on the retinal surface in the control eye injected with PBS (Fig. 9). In eyes treated with reagents, there were no collagen fibrils on the retina. In some cases, few remnants of collagen fibrils of the cortical vitreous were seen; the SEM observations were in line with the B-ultrasonography and indirect ophthalmoscopic findings.

The status of animals on 8^th^ day was as follows: The B scan ultrasonography data suggested occurrence of complete PVD in 5/10 eyes, partial PVD in 3/10 eyes and no PVD in 2/10 eyes treated with reagents (Table 1). Indirect ophthalmoscopy hinted at occurrence of complete PVD in 3/10 eyes, partial PVD in 4/10 eyes and no PVD in 3/10 eyes. SEM data suggests incidence of complete/partial PVD in 8/10 eyes. However, SEM has the disadvantage of examining sections of one half of the eye (while the other half was used for H&E staining), and moreover, only parts of the first half are being observed at a given time, unlike B scan ultrasonography or indirect ophthalmoscopy, which view the entire eye on live animals (Table 1). Overall, intravitreal injection of CBD and RCBD resulted in PVD in 7 out of ten animals, considering all three methods of evaluation.

**Table 1:** Status of PVD in 10 New Zealand rabbits treated with combination of CBD and RCBD in the left eye.

Rabbits N = 10	1	2	3	4	5	6	7	8	9	10
B-Ultrasonography	No PVD	Complete PVD	Complete PVD	Partial PVD	Partial PVD	Partial PVD	Complete PVD	No PVD	Complete PVD	Complete PVD
SEM	Complete PVD	Complete PVD	Complete PVD	No PVD	No PVD	Complete PVD	Complete PVD	Partial PVD	Partial PVD	Complete PVD
Indirect ophthalmoscope	No PVD	Complete PVD	Complete PVD	Partial PVD	No PVD	Partial PVD	Complete PVD	No PVD	Partial PVD	Partial PVD

Statistical evaluation showed that there is a significant effect of the combined treatment of eyes with CBD and RCBD in the context of posterior vitreous detachment when compared to the placebo-treated (PBS injected) eyes. It was found that RCBD + CBD treated eyes are more likely than placebo-treated eyes to have PVD; _χ_2 (2, N = 20) = 13.33, p < 0.001.

#### Light microscopy

Retinal morphology was examined for all the sacrificed animals (n = 20) by light microscopy (H&E staining; Supplementary Fig. S6). Retinal morphology of control eyes matched that of normal eyes, in each of the examined section. Distinct cellular layers of the retina were intact in both, control as well as treated eyes.

## Discussion

It is clear from the recently published Cochrane Review^27,28^ as to why Ocriplasmin is being used only by a handful of surgeons in the clinic. Several factors have been postulated for this, including its highly variable success rate with several reported caveats (age < 65 years, absence of an epiretinal membrane, adhesion diameter <1500 um, phakic status). In addition, adverse events such as photopsias, vitreous floaters and abnormalities of photoreceptor function are also reported^29–34^. Some reports show that ocriplasmin can occasionally result in lasting panretinal structural and functional abnormalities in a subset of eyes, possibly due to enzymatic degradation of laminin and other proteins within the retina. In this connection, it would be reasonable to use non-enzymatic pharmacological methods to effect PVD.

Among the non-enzymatic agents used for this purpose, RGD tripeptide was reported to be useful mainly as an interfactant to disrupt vitreoretinal adhesion^35^. However, the study had some limitations and there has been no further progress in the area of RGD-assisted PVD^9,10^.

Our current study has made use of the ability of RGD based agents (i.e., RGD-fused proteins, rather than RGD tripeptides) to disrupt vireoretinal adhesions, coupled with the use of the collagen binding domain (CBD) of *vibrio mimicus* to liquefy the vitreous gel through unwinding of the triple helical structures of collagen molecules, both as free CBD and as RGD-fused CBD (RCBD).

The CBD derived from the *vibrio mimicus* collagenase has been previously shown to bind to collagen scaffolds, when fused to growth factors^36–38^. It came to our notice recently^39^ that collagen-targeted RGD biomimetic (CBD-RGD) peptide, with the CBD derived from the von Willebrand factor (VWF) and the RGD motif placed in the C-terminus (as opposed to the N-terminal use of RGD in the present study) has been used in the context of osteogenesis, to supplement bone morphogenetic protein (BMP) for induction of osteoblastic differentiation. Our study presents a different construct, CBD, derived from *vibrio mimicus* collagenase, ~12 kDa protein, rather than only the collagen binding section of the von Willebrand factor (VWF), composed of 15 amino acids, used by Visser *et al*.^39^, for which the estimated half- life by the ProtParam tool (ExPASy; SIB Bioinformatics Resource Portal) is only a couple of hours. The construct RCBD, on the other hand, was found to be stable for over 48 hours, following incubation in the presence of vitreous sample. Also, the rationale of using the combination of CBD and RCBD and the particular context of PVD are different and novel, in addition to the novelty of use of a collagen-destabilizing domain, rather than merely a collagen binding domain. Even so, it is satisfying that another group has considered fusing the two components in a therapeutic agent, since this suggests an intrinsic merit in the basic idea of exploring the use of such a fusion.

It would seem that instead of using a combination of RCBD and CBD, RCBD alone could be used to achieve the dual roles of CBD and RGD tripeptide, since RCBD incorporates both the CBD and the tripeptide. However, we chose to use the combination of two reagents for the following reasons: (1) An RGD tripeptide would be likely to be less stable to proteolysis and degradation than an RGD extension on a protein (any protein) for reasons relating to differential steric restriction of the approach of proteases to the peptide bonds in the RGD section of an RGD-protein fusion, as opposed to what would apply to free RGD peptide. (2) If RGD were to be fused to a protein (any protein), for reasons of making it more proteolytically-stable within such a fusion, it would appear to be more beneficial to fuse RGD to a protein (such as CBD) which brings an alternative route to addressing the original problem being addressed through the use of RGD. (3) The problem inherent in fusing RGD to CBD and using this fusion reagent alone is that it is not necessary that the RGD and CBD components are required to the same extent, since clearly the CBD could function to destabilize collagen triple helices and keep them in an unwound (monomeric or liquefied) state throughout the vitreous humor, whereas the RGD would only be required to act in the vicinity of integrins, on cell surfaces. (4) Therefore, we decided to use a combination of CBD and RCBD, to both (a) deliberately maintain a significant molar excess of CBD availability over RGD availability, and (b) maintain RGD in a protein-fused form (RCBD) to improve its stability to proteolytic degradation, and also allow the fusion to perform both its functions, individually as well as simultaneously (at cell surfaces). It may be noted that use of RCBD alone would allow the excessive numbers of integrins present on cell surfaces to titrate away and sequester the bulk of the RCBD population, and keep it away from destabilizing collagen in the vitreous. It was conceived that the use of CBD and RCBD in a suitable combination would allow collagen to remain structurally destabilized throughout the vitreous and in the vicinity of the ILM, and also allow the RGD component of RCBD to bind to integrins.

As it turned out, adhesion of cells to the collagen matrix was less when cells were incubated with RCBD as compared to CBD-treated cells. These results can be explained as follows: Collagen type 1 has 2 motifs for binding to cells through the integrin receptors: the sequence stretch, “GFOGER” and the tripeptide, “RGD” motifs (2 in the α1 chain and 3 in the α3 chain)^40^. ARPE-19 cells can bind to the collagen surface via both these motifs^41,42^. These integrin-binding motifs remain largely unexposed in the native collagen triple helix, with the resultant weak binding of cells to collagen (Fig. 5A). It has been shown that when the collagen triple helix loosens up, for example as a result of denaturation, then these motifs become more accessible to cell surface integrins^43^. It is to be expected therefore, that when CBD binds to the collagen triple helix, it will result in unwinding of the helix (as suggested by our data explained above), leading to increased exposure of the integrin-binding motifs, and the consequent increase in adhesion of cells to collagen, as compared to cells in the absence of CBD (Fig. 5B). The RCBD construct includes the CBD domain which is expected to increase the adhesion of cells to collagen; however, RCBD also has the tripeptide, RGD, which has the ability to bind to cell surface integrins^18^, thereby posing a competition to the interaction of cell surface integrins with collagen. This blocking of integrins with the RGD motif is therefore expected to cause a reduction in interaction (adhesion) of cells with collagen. Overall, therefore, it is to be anticipated that RCBD-treated cells will have less adhesion to collagen matrix as compared to CBD, because of the dual, opposing actions of CBD and RGD in RCBD (Fig. 5C).

Out of a total of 10 rabbits examined in response to treatment with CBD and RCBD, only 2 animals did not show any PVD. None of the controls showed any PVD. There were no signs of retinal detachment, cataract or haemorrhage or fibrin formation in the anterior chamber in eyes treated with CBD and RCBD, on day 8. As such, no retinal toxicity was seen as judged by fundus examination as well as H&E staining of retinal sections, even on day 15 following injection with reagents. However, w e acknowledge that our study lacks in a functional evaluation of the retina (by methods such as ERG).

Very few non-enzymatic reagents have been tried so far for the purpose of vitreolysis^9,10^. Among these, RGD peptide-based vitrectomy^35^ and the urea-based vitreosolve^44^ are two examples. In the former case, the initial results were encouraging, but no further studies have been reported in the last 17 years. In the case of the latter, the results of clinical trials turned out to be disappointing.

Enzymatic reagents such as Nattokinase, tissue plasminogen activator and urokinase have the disadvantage that the dosing is not precise, being dependent on amounts of plasminogen available in the vitreous and the state of the blood-retinal barrier^45^. Plasmin is not available commercially, and the preparation of autologous plasmin requires sterility, and is cumbersome; moreover, the molecule is unstable, and the activity is not reproducible^46,47^. In light of this, it would seem logical to devise new non-enzymatic and non-toxic reagents for the purpose of liquefaction of the vitreous gel as well as posterior vitreous separation, to obviate the criticality of dosing in situations involving toxic reagents.

When we compared our data with previously published results^46,48,49^ in terms of percentage of PVD achieved with use of pharmacological reagents, it emerged that combined treatment with CBD and RCBD results in comparable liquefaction as well as separation of posterior vitreous as reported by others in the field, with the added benefit of non-toxicity and safety to the retinal tissues, which is to be expected owing to the nature (non-enzymatic) of the reagents.

It may be noted that use of any pharmacological vitreolysis reagent runs the risk of introducing additional floaters, by causing inhomogeneity of refractive indices; however, the advantage would appear to outweigh the disadvantage.

Overall, our results demonstrate the safety and efficacy of using a combined dose of CBD and RCBD in inducing PVD.

## Methods

### Ethics statement

Informed consent for use of vitreous was obtained from patients undergoing retinal re-attachment surgeries. Institutional Review Board of the institute (PGIMER) accorded approval of the study protocol (PGI/IEC/2013/2009–10). The guidelines of the Declaration of Helsinki were observed.

### Cloning of CBD and RCBD

Using the *vibrio mimicus* collagenase (VMC) gene as a template^16^, the collagen-binding domain or CBD (amino acid stretch between L528 and T628) was amplified with primers having restriction sites BamH1 and Sac1 incorporated in the forward and reverse primers respectively (Forward: 5′GGCCCGGATCCTTGGTACTGTCTCGACCAGGG3′; Reverse:5′ATGTAAGAGCTC TCATGTAAAGATCGGCGTCGCATCTCC3′). The 300 bp long PCR product was digested by the two enzymes, and ligated into pQE30 vector, also cut with the same two enzymes, to generate insert-compatible sites for ligation. The ligation mix was transformed into XL1-Blue competent cells and the positive clones were screened out using colony PCR, and verified by sequencing.

Since the pQE30 vector comprises of Met start codon and RGS-His epitope downstream of the promoter region, therefore, cloning any gene within the MCS site of the vector results in a stretch containing MRGS-6His tag at the N-terminal of the protein. We re-engineered the pQE30 vector and inserted 3 nucleotides (coding for aspartic acid, ‘D’) within the MRGS-His region (as shown in Supplementary Fig. S1). Amino acid ‘D’ was inserted following the MRG amino acids, to give the final stretch of residues as MRGD-6His. This re-engineered vector thus had an intact MCS site and other features. Next, the BamH1 and Sac1 digested CBD amplicon was inserted into the modified pQE30-RGDS vector, which had also been similarly digested with the two enzymes. Protein expression was done by transforming the construct in M15(pREP4) cells. The expression and purification of CBD as well as RCBD was carried out as described earlier^50^. The resulting recombinant protein thus consisted of an RGD motif in the N-terminal and it was named as RCBD or the fusion of RGD and CBD.

### Assessment of stability of CBD and RCBD in cadaver vitreous by western blotting

For this experiment, vitreous from donor eyes (obtained from the Eye Bank, PGIMER), was used, from which healthy corneas had been used for keratoplasty. The vitreous was mechanically liquefied and test reagents were added to it in a ratio of 1:1; tubes were kept at room temperature. Final concentrations of the recombinant test reagents in the presence of vitreous were as follows: CBD and RCBD − 0.25 mg/ml; combination of CBD and RCBD − 0.125 mg/ml each. 40 µl of the sample from each tube was taken out at 12 h intervals up to 48 h. Then 10 μl of 5X reducing dye was added and the sample was boiled. Samples were run on 12% SDS PAGE. The proteins were transferred onto nitrocellulose membrane, blocked with 5% skimmed milk for 2 h at 37 °C, followed by probing with mouse anti-His-HRP conjugated antibody (Sigma #A7058). The signal was visualized by ECL reagent and captured on the FluorChem E Imaging System (Protein Simple). For quantitative analysis, bands were measured densitometrically using ImageJ (https://imagej.nih.gov/ij/; NIH, USA) software. Amount of protein at 0 h was taken as control, and the data was plotted as % of intact protein remaining with reference to 0 h.

### Immunofluorescence staining of ARPE-19 cells for β1 integrin

For this experiment, 2 male patients (aged 32 and 67 years) with pseudophakic rhegmatogenous retinal detachments (RRD) undergoing pars plana vitrectomy, were chosen for collection of vitreous and subretinal fluid during the surgery. Around 1 million ARPE-19 cells were cultured in the presence of vitreous/subretinal fluid on a cover slide in a 24-well plate, as described previously^51^. The next day, 30 µM RCBD was added to cells and incubated at 37°C for 4–5 h; cells cultured in the absence of RCBD were taken as control. Cells were fixed using 4% paraformaldehyde at room temperature for 10 min, washed with PBS, and incubated in the presence of anti-β1 integrin antibody (Sigma, Prod#I8638; 1:100 dilution in 1% BSA in PBS) overnight at 4°C. FITC tagged secondary antibody (Invitrogen Alexa Flour 488 goat anti-mouse IgG cat#A11001) was added and incubated for 1–2 h at 4°C. Nuclear dye DAPI was used (1:20 K dilution) for 15 min and washed gently. Cover slips were mounted on slides and images were taken on the confocal microscope (Olympus FV1000).

### Assessment of ARPE-19 cell viability by MTT assay

MTT assay was carried out to test viability of ARPE-19 cells cultured with different doses of CBD/RCBD. Approx. 10,000 ARPE-19 cells (in 200 µl DMEM HG plus 10% FBS media) were seeded per well in a 96 well plate. Cells were incubated overnight (37°C, 5% CO_2_), and serum-starved for 5–6 h, followed by addition of MTT to (final working concentration 0.5 mg/ml) to each well; incubation was done for 3–4 h to allow the formation of crystals. Crystals were dissolved in 100 µl DMSO per well, and absorbance was measured at 570 nm with a reference wavelength at 630 nm. Cells in Serum-Free well (SF) were taken as a reference control. Statistical analysis was performed on three independent sets of experiments.

### CD spectroscopy of collagen

CD spectroscopy was carried out according to previously reported protocol^52^ using Chirascan CD Spectrophotometer equipped with a peltier based heating arrangement; spectra were recorded from 200 nm to 250 nm with 1 nm bandwidth. Briefly, 3 mg/ml type 1 collagen stock (Advanced Biomatrix, cat#5007) in 0.1 N HCL was diluted to 1.5 mg/ml with 2X collagen neutralization buffer (final concentration of collagen, 0.25 µM) and was kept in ice for 10 minutes. The collagen solution (in PBS) was incubated with a range of concentrations of CBD and RCBD (1.25 µM −10 µM) at 30 °C up to 3 h. Collagen solution in PBS without any other added proteins was used as control. Solutions of corresponding concentrations of CBD and RCBD alone (in PBS) were used for baseline measurements, to be subtracted from the respective collagen spectra. Average of three scans were taken for each sample, and spectra were plotted as MRE (Mean Residual Ellipticity) vs wavelength using mean residue weight (MRW) of 91.2 for collagen type 1.

### Structural analysis of VMC variants with circular dichroism spectroscopy

0.2 mg/ml of CBD, RCBD or VMC in PBS was used to record CD spectra of each of these proteins, as described above.

### Solubilization of polymerized collagen by CBD and RCBD

Commercially sourced collagen type 1 (Advanced Biomatrix, cat#5007) was polymerized in the presence of neutralizing buffer, in order to mimic the polymeric collagen network present in the vitreous gel. This was followed by treatment with two different concentrations (20 µM and 40 µM) of CBD and RCBD individually, and a combination of both proteins, CBD and RCBD (10 µM CBD and 10 µM RCBD; 20 µM CBD and 20 µM RCBD). Tubes were incubated overnight at room temperature; the supernatant from each tube was run on SDS-PAGE to assess the released monomeric soluble collagen (signifying liquefaction of collagen), in terms of increased intensity of collagen bands (α1, α2, β) observed after coomassie staining. For quantitative analysis, the α1 bands of samples treated or untreated with proteins (control samples) were measured densitometrically using ImageJ (https://imagej.nih.gov/ij/; NIH, USA) software. Amount of soluble collagen was plotted as the ratio of intensity of α1 band in treated vs control samples.

### Cell adhesion assay

Cell adhesion assay was carried out using ARPE-19 cells on collagen type 1 coated surface (0.1 mg/ml collagen type 1; Advanced Biomatrix, cat#5007). Wells were blocked with 2% BSA for 2–3 h at room temperature. Separately ARPE-19 cells (around 25 K) were incubated with different doses of CBD and RCBD (15 µM and 30 µM of CBD or RCBD and a combination dose of 15 µM CBD along with 15 µM RCBD), or in the absence of reagents (control), and seeded onto the wells. Incubation was done at 37 °C in a CO_2_ incubator for 1 h. Wells were washed extensively with PBS to remove unattached cells, and stained with crystal violet (0.25% crystal violet in 10% ethanol), followed by washing with PBS again to remove the excess stain; imaging was done on inverted light microscope. For quantitative measurement, the stain was dissolved in 50% ethanol in 0.1 M sodium dihydrogen solution and readings were taken at 570 nm wavelength.

### Assessment of vitreolysis in goat eyes by rheometry

Goat eyeballs were procured from local slaughterhouse within 2–3 h of sacrifice. After an initial inspection of the eyeballs, and multiple washings with PBS, reagents were injected mid-vitreous (0.25 ml) using a 30 gauge needle; control eyes received an equivalent volume of PBS alone. Other vitreolysis controls included a negative control (BSA) and a positive control (Collagenase IV; Sigma, cat#C5138). Small sized eye balls (n = 3; vitreous volume ~4 ml) were used; CBD and RCBD were used at a concentration of 0.3 mg each; a combination dose, CBD + RCBD (0.3 mg) was also used. Following intravitreal injection, eyeballs were incubated for 5 h at 30°C and using a 7 mm biopsy punch, vitreous was punched out from multiple regions of the vitreous body, including mid-vitreous and periphery regions, to remove location-specific bias in sampling, and placed on a rheometer. Only those data points showing linear viscoelastic behaviour were included for plotting the storage modulus (G’) graph. All the rheological studies were done on Anton-Paar (MCR 102) instrument, as described earlier^26^. The temperature of the sample stage was set at 20°C; shear strain amplitude (γ_0_) was set at 3% with an oscillatory frequency sweep, ω = π/5 to 2π rad/sec, to obtain a linear viscoelastic behavior. A portion of the vitreous (7 mm biopsy punch) was kept on the sample stage. The detector (measuring cone) (8 mm, CP08-1, part no. 80601) was lowered down up to within 2 mm of the sample so that it barely touched the vitreous sample, and it was kept in this position for approximately 1 min. The gap between the measuring cone and stage were then set in automatic mode. Storage modulus (G’), loss modulus (G”) and dumping factor or loss factor (tan δ = G”/G’) were measured for each sample, to determine the linear viscoelastic region. Three independent experiments were carried out, and for each concentration of a reagent, a total of three eyeballs were evaluated.

### Scanning electron microscopy

Scanning electron microscopy was carried out for *ex vivo* experiments (goat eye balls) as well as *in vivo* experiments (post-sacrifice of rabbit eye balls).

Fixation was done using 4% paraformaldehyde and 1% glutaraldehyde for goat eyes balls and 1% paraformaldehyde and 1.25% glutaraldehyde for rabbit eye balls (in 0.08 M Sodium Cacodylate buffer, pH 7.4) at 4°C for 2–3 days. Post fixation, the anterior section was taken out and cut into four sections carefully with a sharp scalpel, and washed two times with cacodylate buffer. Samples were gradually dehydrated with an increasing percentage of ethanol (40%, 50%, 60%, 70%, 80%, 90% for 20 min each, and then three times with 100% ethanol for 20 min each). Samples were dried using Critical Point Drying (CPD) and coated with platinum. Images were taken in the SEM microscope (Jeol, JSM-IT300, Japan).

### OCT of *ex vivo* goat eye balls

OCT of goat eye balls was performed as reported earlier^53^ with slight modifications. In brief, eye balls were placed in a customized eye ball holder at a height slightly above the chin rest of the OCT system (Spectralis; Heidelberg Engineering, Heidelberg, Germany). The instrument was first adjusted to ensure focusing of the optic disc, following which horizontal and vertical raster line scans were taken. Lubricant eye drops were constantly used over the corneal surface to prevent its drying.

### *In vivo* experimental studies

All animal experiments were approved by the Institute Animal Ethics Committee, PGIMER Chandigarh (Ref No 97/IAEC/678). The animals were treated in accordance with the ARVO statement for the use of animals in vision and ophthalmic research. The study was carried out on New Zealand Albino Rabbits (M/F, 5–6 months old, 2–2.5 Kg weight). One eye (left) of each animal (n = 10) received a combination dose of CBD & RCBD (150 µg each; 0.1 ml), while the contralateral eye (right) received only PBS and was treated as control. A brief experimental outline is given in Supplementary Fig. S5.

Animals were anesthetized with subcutaneous injection of Ketamine (35 mg/Kg body weight) and Xylazine (5 mg/Kg body weight). Before the injection, eyes of each animal were examined by an ophthalmologist for confirmation of normal ocular health. A drop of betadine was instilled into the eye followed by administration of intravitreal injection. Reagent/PBS was injected through a 30 gauge needle inserted to a point which is 1.5 mm posterior from the limbus in the superotemporal quadrant, in the mid vitreous cavity. Immediately after the syringe was taken out, betadine drops were given, followed by moxifloxacin eye drops.

The day of intravitreal injection was counted as day zero and so on. Clinical follow-up was carried out by two trained ophthalmologists independently, in a blinded fashion, on days 1, 4 and 8 by means of indirect ophthalmoscopy (with 20 diopter lens) and B-ultrasonography (HiScan Touch; Optikon, Italy). Fundus pictures were taken on Spectralis (Heidelberg Engineering, Heidelberg, Germany). Ultrasonography was done using the A and B scan modes, and the horizontal and vertical axial scans were taken to view the optic nerve head. B scan ultrasonography of any membrane either floating in the vitreous cavity or attached to the optic disc was noted in serial scans.

### Histologic examination

Post fixation, the rabbit eye globes were hemisected at roughly the horizontal meridian using a sharp scalpel; one half of the eye globe was processed for SEM, while the other half was used for histological examination. The tissue was kept at 4°C overnight in phosphate buffer saline, followed by dehydration through a graded ethanol series and propylene oxide, and embedded in paraffin. Horizontal sections (5-mm thickness) were made through the optic nerve head and staining was done with hematoxylin and eosin. At least three sections were evaluated; in each case, the entire field was scanned by the pathologist (blinded with respect to sample identity) for any morphological differences in the retinal sections.

### Statistical analysis

Results are expressed as mean ± SD. One-way Anova test was used for analysis of data. A p value < 0.05 was considered to be significant.

For animal data, results were compared using analysis of cross-table and Pearson w2 test. p < 0.05 was considered statistically significant.

## Supplementary information

Supplementary Information.
