# Author Correction: Induction of posterior vitreous detachment (PVD) by non-enzymatic reagents targeting vitreous collagen liquefaction as well as vitreoretinal adhesion

**DOI:** 10.1038/s41598-020-69093-w

**Published:** 2020-07-16

**Authors:** Mithun Santra, Maryada Sharma, Deeksha Katoch, Sahil Jain, Uma Nahar Saikia, Mangat R. Dogra, Manni Luthra-Guptasarma

**Affiliations:** 1 Department of Immunopathology, Postgraduate Institute of Medical Education and Research, Chandigarh, 160012 India; 2 Present Address: Department of Otolaryngology and Head & Neck Surgery, Postgraduate Institute of Medical Education and Research, Chandigarh, 160012 India; 3 Department of Ophthalmology, Postgraduate Institute of Medical Education and Research, Chandigarh, 160012 India; 4 Department of Histopathology, Postgraduate Institute of Medical Education and Research, Chandigarh, 160012 India

Correction to: *Scientific Reports* 10.1038/s41598-020-64931-3, published online 19 May 2020

The below paragraph contains references to articles hypothesising the same concept which has now been experimentally demonstrated; these were omitted from the Discussion section of the Article.

“The concept that we experimentally demonstrated (combination pharmacologic vitreolysis achieved by concomitant performance of vitreous gel liquefaction and vitreous-retinal dehiscence) has been previously theoretically proposed^1,2^. Further, the possibility of combining enzymatic reagents for induction of posterior vitreous detachment (PVD), using hyaluronidase for liquefaction and plasmin for dehiscence has been successfully tested in diabetic rats^3^”.
