# Supplementary material for: Induction of posterior vitreous detachment (PVD) by non-enzymatic reagents targeting vitreous collagen liquefaction as well as vitreoretinal adhesion
Source: Sci Rep. 2020 May 19;10:8250. doi: 10.1038/s41598-020-64931-3 (PMC7237681; doi:10.1038/s41598-020-64931-3)
Supplement: Supplementary file 1 — Supplementary Information. [file 41598_2020_64931_MOESM1_ESM.pdf]

# Induction of posterior vitreous detachment (PVD) by non-enzymatic reagents targeting vitreous collagen liquefaction as well as vitreoretinal adhesion

**Mithun Santra[1], Maryada Sharma[1$], Deeksha Katoch[2], Sahil Jain[2], Uma Nahar Saikia[3], Mangat R Dogra[2], Manni Luthra-Guptasarma[1]**

[1] Department of Immunopathology, [$]Department of Otolaryngology and Head & Neck surgery (Current affiliation), [2] Department of Ophthalmology, [3]Department of Histopathology, Postgraduate Institute of Medical Education and Research, Chandigarh, 160012

.

*Running title : Targeting collagen triple helix and integrin in PVD

To whom correspondence should be addressed: Manni Luthra-Guptasarma, Department of Immunopathology, Research Block A, Postgraduate Institute of Medical Education and Research, Sector 12, Chandigarh, 160012.

*Tel: 91-172-2755196; Fax: 91-172-2744401*

*Email:guptasarma.manni@pgimer.edu.in; mguptasarma@yahoo.com*

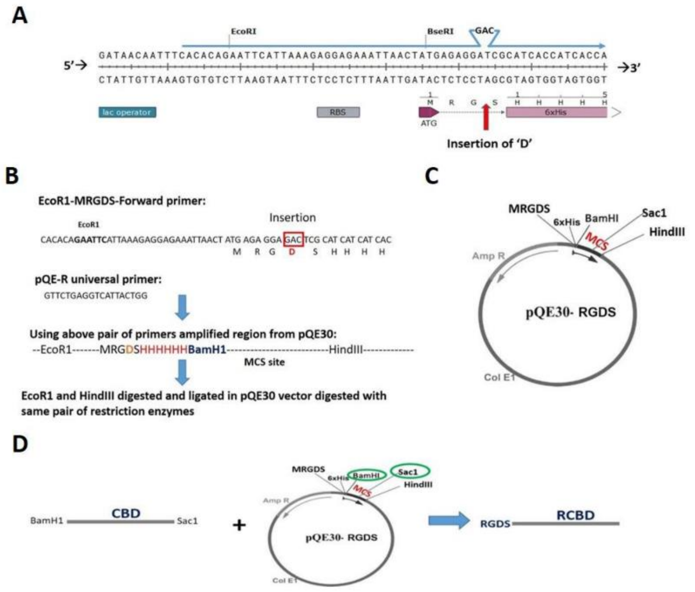


**Supplementary Fig S1**: Diagrammatic representation of the cloning strategy for RCBD.

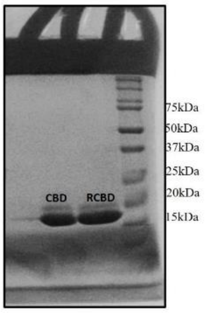


**Supplementary Fig S2**: Un-cropped version of the SDS-PAGE profile of gel displayed in Fig 1B.

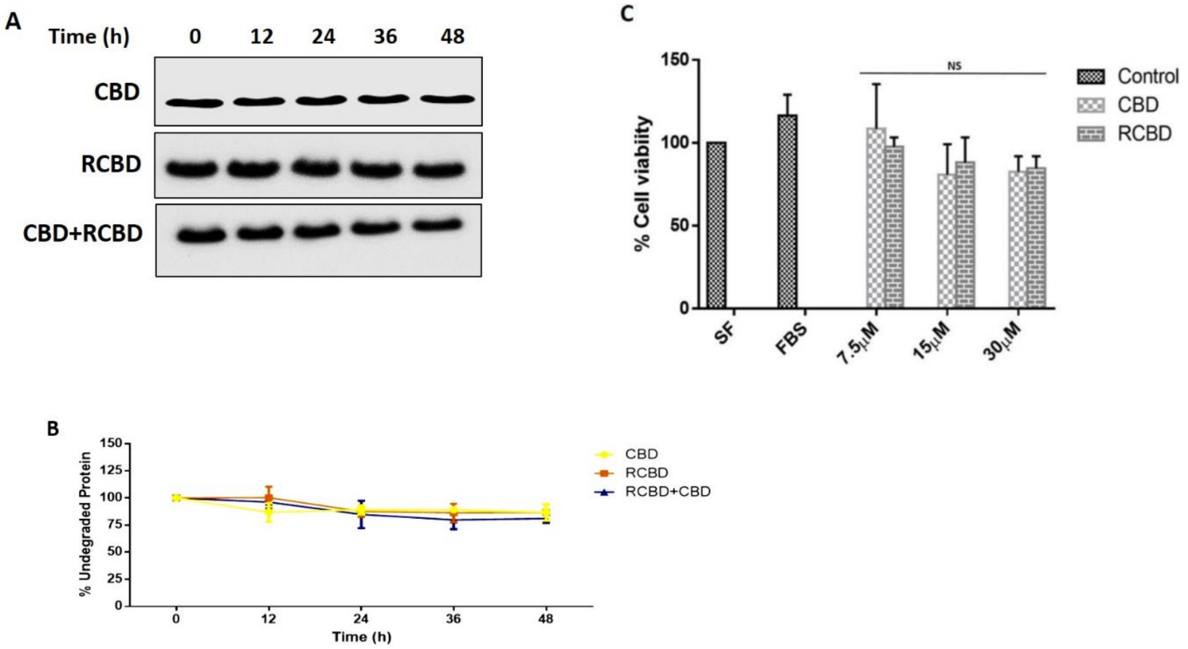


**Supplementary Fig S3:** (Panel A): Evaluation of degradation rate of the proteins CBD and/or RCBD, following incubation of the proteins in human donor vitreous for up to 48 h; western blotting using HRP labelled mouse anti-His antibody was done to detect the intact protein. Individual blots were developed for CBD, RCBD and CBD plus RCBD respectively, and cropped images (shown in separate boxes) are presented here. The raw western blot results (uncropped) are available in Supplementary Figure S4. (Panel B): Assessment of viability of ARPE-19 cells (MTT assay); cells were incubated overnight with different doses of the reagents. ARPE-19 cells were viable up to 30 µM of CBD or RCBD (NS: not significant)

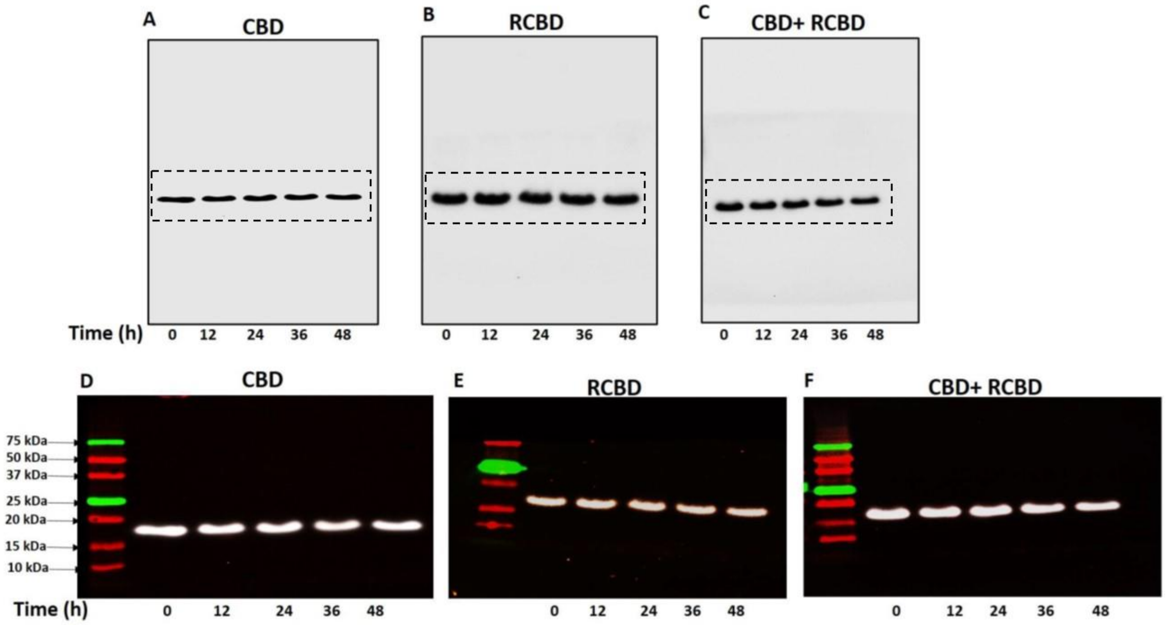


**Supplementary Fig S4:** Uncropped version of the same blots displayed in Supplementary Fig S3A. Panels A-C: Inverted images of the blots (using the inverted image display option in AlphaView software available on the ProteinSimple instrument, to see dark bands on a light background). The highlighted, dashed areas are the regions used for cropping; Panels D-F: Original, uncropped images of the blots, shown to include the molecular weight markers.

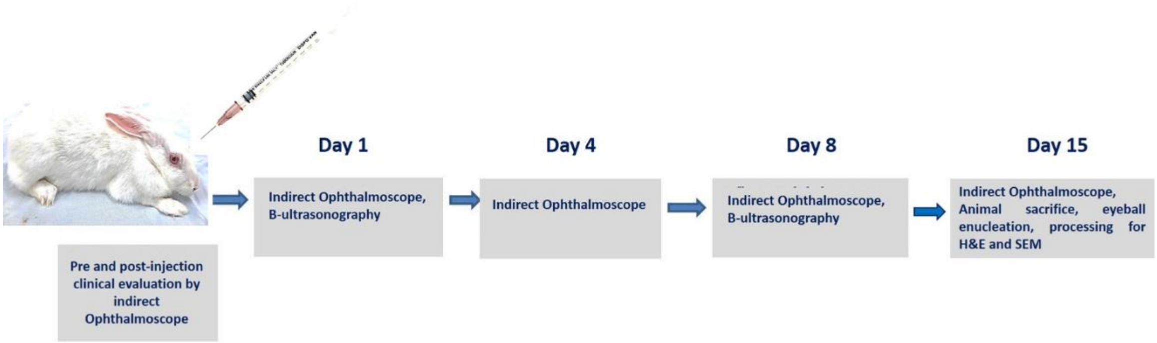


**Supplementary Fig S5:** Timeline of animal experiments.

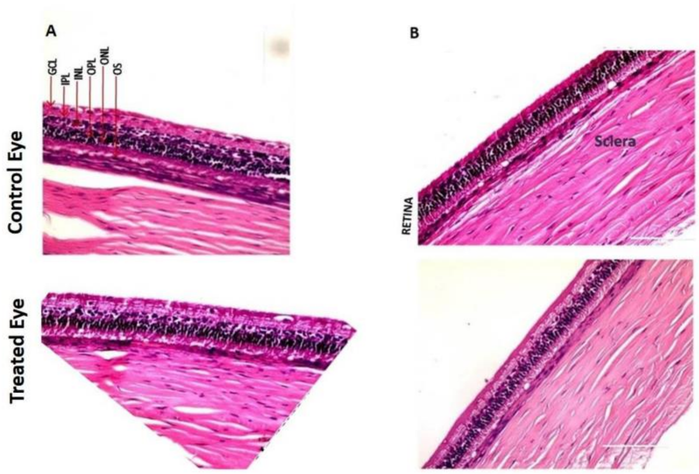


**Supplementary Fig S6:** Light micrographs of retinal cross-section (H&E staining). The retinal morphology of eyes from two representative animals (A and B) injected with PBS (control) or combination of CBD and RCBD (treated animals). No significant differences were observed in the histology of the retina between the control and treated animals by the end of this study (15 days). (**Scale bar 100 µm or 40X magnification, **GCL, ganglion cell layer; IPL, inner plexiform layer; INL, inner nuclear layer; OPL, outer plexiform layer; ONL, outer nuclear layer; OS, outer segments of photoreceptors)

Supplementary Table 1: Results of clinical follow-up of rabbits on day 8, following treatment with RCBD plus CBD

| Rabbits | | 1 | | 2 | | 3 | | 4 | | 5 | | 6 | | 7 | | 8 | | 9 | | 10 | |
|---|---|---|---|---|---|---|---|---|---|---|---|---|---|---|---|---|---|---|---|---|---|
| RCBD +CBD group | | Treated eye | Control Eye | Treated eye | Control Eye | Treated eye | Control Eye | Treated eye | Control Eye | Treated eye | Control Eye | Treated eye | Control Eye | Treated eye | Control Eye | Treated eye | Control Eye | Treated eye | Control Eye | Treated eye | Control Eye |
| Day 1 | M. Grade | 2 | 1 | 2 | 1 | 1 | 1 | 1 | 1 | 1 | 1 | 1 | 1 | 1 | 1 | 1 | 1 | 1 | 1 | 1 | 1 |
| | AC Fibrin | + | - | + | - | - | - | + | - | - | - | - | - | - | - | - | - | - | - | - | - |
| | AC Hem | - | - | - | - | - | - | - | - | - | - | - | - | - | - | - | - | - | - | - | - |
| | Lens Opacity | - | - | - | - | - | - | - | - | - | - | - | - | - | - | - | - | - | - | - | - |
| | Vit Hem | - | - | - | - | - | - | - | - | - | - | - | - | - | - | - | - | - | - | - | - |
| | R.D. | - | - | - | - | - | - | | | | | - | - | - | - | - | - | - | - | - | - |
| Day 4 | M Grade | 1 | 1 | 1 | 1 | 2 | 1 | 1 | 1 | 1 | 1 | 1 | 1 | 1 | 1 | 1 | 1 | 1 | 1 | 1 | 1 |
| | AC Fibrin | - | - | - | - | + | - | - | - | - | - | - | - | - | - | + | - | - | - | - | - |
| | AC Hem | - | - | - | - | - | - | - | - | - | - | - | - | - | - | - | - | - | - | - | - |
| | Lens Opacity | - | - | - | - | - | - | - | - | - | - | - | - | - | - | - | - | - | - | - | - |
| | Vit Hem | - | - | - | - | - | - | - | - | - | - | - | - | - | - | - | - | - | - | - | - |
| | R.D. | - | - | - | - | - | - | - | - | - | - | - | - | - | - | - | - | - | - | - | - |
| Day 8 | M. Grade | 1 | 1 | 1 | 1 | 2 | 1 | 1 | 1 | 1 | 1 | 1 | 1 | 1 | 1 | 1 | 1 | 1 | 1 | 1 | 1 |
| | AC Fibrin | - | - | - | - | - | - | - | - | - | - | - | - | - | - | - | - | - | - | - | - |
| | AC Hem | - | - | - | - | - | - | - | - | - | - | - | - | - | - | - | - | - | - | - | - |
| | Lens Opacity | - | - | - | - | - | - | - | - | - | - | - | - | - | - | - | - | - | - | - | - |
| | Vit Hem | - | - | - | - | - | - | - | - | - | - | - | - | - | - | - | - | - | - | - | - |
| | R.D. | - | - | - | - | - | - | - | - | - | - | - | - | - | - | - | - | - | - | - | - |

** **M Grade;** Media Grade (Vitreous Clarity), Grade 1 is the normal clarity. **AC Fibrin;** Anterior Chamber Fibrin Formation. **AC Hem**; Anterior Chamber Hemorrhage. **Lens Opacity;** Cataract/ Lens clarity. **Vit Hem;** Vitreous Hemorrhage. **R. D.;** Retinal Detachment
